# A cross‐tissue transcriptome‐wide association study identifies novel susceptibility genes for atrial fibrillation

**DOI:** 10.1002/joa3.70097

**Published:** 2025-05-22

**Authors:** Yalin Yuan, Xin Zheng, Wenjing Zhang, Zhaoyu Ren, Bin Liang

**Affiliations:** ^1^ Shanxi Medical University Taiyuan Shanxi China; ^2^ Department of Cardiovascular Medicine Second Hospital of Shanxi Medical University Taiyuan Shanxi China

**Keywords:** atrial fibrillation, colocalization, cross‐tissue TWAS, Mendelian randomization, UTMOST

## Abstract

**Background:**

Atrial fibrillation (AF), the most common cardiac arrhythmia, has been linked to numerous loci identified by genome‐wide association studies (GWAS). However, the causal genes and underlying mechanisms remain unclear.

**Methods:**

We conducted a cross‐tissue transcriptome‐wide association studies (TWAS) using the unified test for molecular signatures (UTMOST), integrating genetic data from the FinnGen R11 cohort (287 805 individuals) with gene expression profiles from the genotype‐tissue expression (GTEx) project. To enhance reliability, we applied functional summary‐based imputation (FUSION), fine‐mapping of causal gene sets (FOCUS), and multi‐marker analysis of GenoMic annotation (MAGMA) for gene prioritization, followed by Mendelian randomization (MR) and colocalization analyses. GeneMANIA was used to explore gene functions.

**Results:**

By integrating four TWAS approaches, this study identified five novel susceptibility genes significantly associated with AF risk. MR analysis further revealed that the gene expression levels of FKBP7, CEP68, and CAMK2D were positively associated with AF risk, while SPATS2L exhibited a significant protective effect. Colocalization analysis demonstrated that CEP68 and SPATS2L share causal variants with AF. Through comprehensive evaluation of multidimensional functional annotations and existing biological evidence, this study highlighted SPATS2L and CEP68 as potential functional candidate genes in AF pathogenesis.

**Conclusions:**

This cross‐tissue TWAS identified five novel AF susceptibility genes (CAMK2D, SPAST2L, CEP68, FKBP7, and SHRMOO3). Elevated expression of FKBP7, CEP68, and CAMK2D increases AF risk, while SPATS2L showed a protective effect, with colocalization analysis implicating CEP68 and SPATS2L as prioritized candidates. The integration of multi‐omics approaches effectively unravels AF's genetic mechanisms.

## Graphical abstract

This cross‐tissue transcriptome‐wide association study identified five novel AF susceptibility genes (CAMK2D, SPAST2L, CEP68, FKBP7, and SHRMOO3). Elevated expression of FKBP7, CEP68, and CAMK2D increases AF risk, while SPATS2L shows a protective effect, with colocalization analysis implicating CEP68 and SPATS2L as prioritized candidates. This study highlights SPATS2L and CEP68 as potential functional candidate genes in AF pathogenesis.

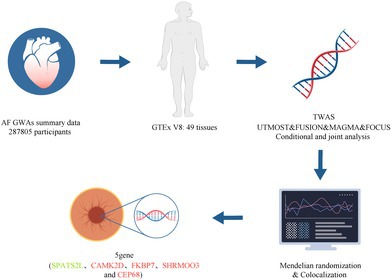

## 1 INTRODUCTION

Atrial fibrillation (AF) is the most common sustained arrhythmia, characterized by disorganized electrical activity in the atria, leading to complications such as stroke, heart failure, and reduced quality of life.^1^ Despite extensive research, the genetic underpinnings of AF remain incompletely understood.

Familial studies have shown that genetics plays a substantial role in AF onset.^2^ Genome‐wide association studies (GWAS) have identified over 100 risk loci associated with AF; however, these loci collectively explain only a small fraction (6.4%) of its heritability.^3^ Most GWAS signals reside in noncoding regions, suggesting that transcriptional regulation plays a crucial role in AF susceptibility,^4^ This highlights the need for alternative approaches to uncover the functional mechanisms linking genetic variants to AF pathogenesis.

Transcriptome‐wide association studies (TWAS) have emerged as a powerful tool to bridge this gap by integrating gene expression data with genetic variation.^5^ Unlike GWAS, which primarily focuses on genetic variants, TWAS leverages expression quantitative trait loci (eQTL) data to identify genes whose expression levels are associated with disease risk. This approach has successfully identified novel susceptibility genes for various complex diseases, including migraines,^6^ rheumatoid arthritis,^7^ and lung cancer.^8^ However, most TWAS analyses are limited to single‐tissue gene expression data, potentially overlooking the regulatory effects of eQTLs across multiple tissues.^5^

In this study, we performed a cross‐tissue TWAS to identify novel susceptibility genes for AF by integrating eQTL data from the genotype‐tissue expression (GTEx) project with AF GWAS data from the FinnGen R11 dataset. We employed multiple analytical techniques, including functional summary‐based imputation (FUSION),^9^ fine‐mapping of causal gene sets (FOCUS),^10^ and multi‐marker analysis of GenoMic annotation (MAGMA), to ensure the robustness of our findings. Additionally, we conducted Mendelian randomization (MR) and colocalization analyses to validate the causal relationships between gene expression and AF risk. This is the first cross‐tissue TWAS integrating multi‐omics approaches to identify novel AF susceptibility genes, addressing the limitations of single‐tissue analyses.

## 2 MATERIALS AND METHODS

### 2.1 Study design and data sources

#### 2.1.1 Study design

**FIGURE 1:**
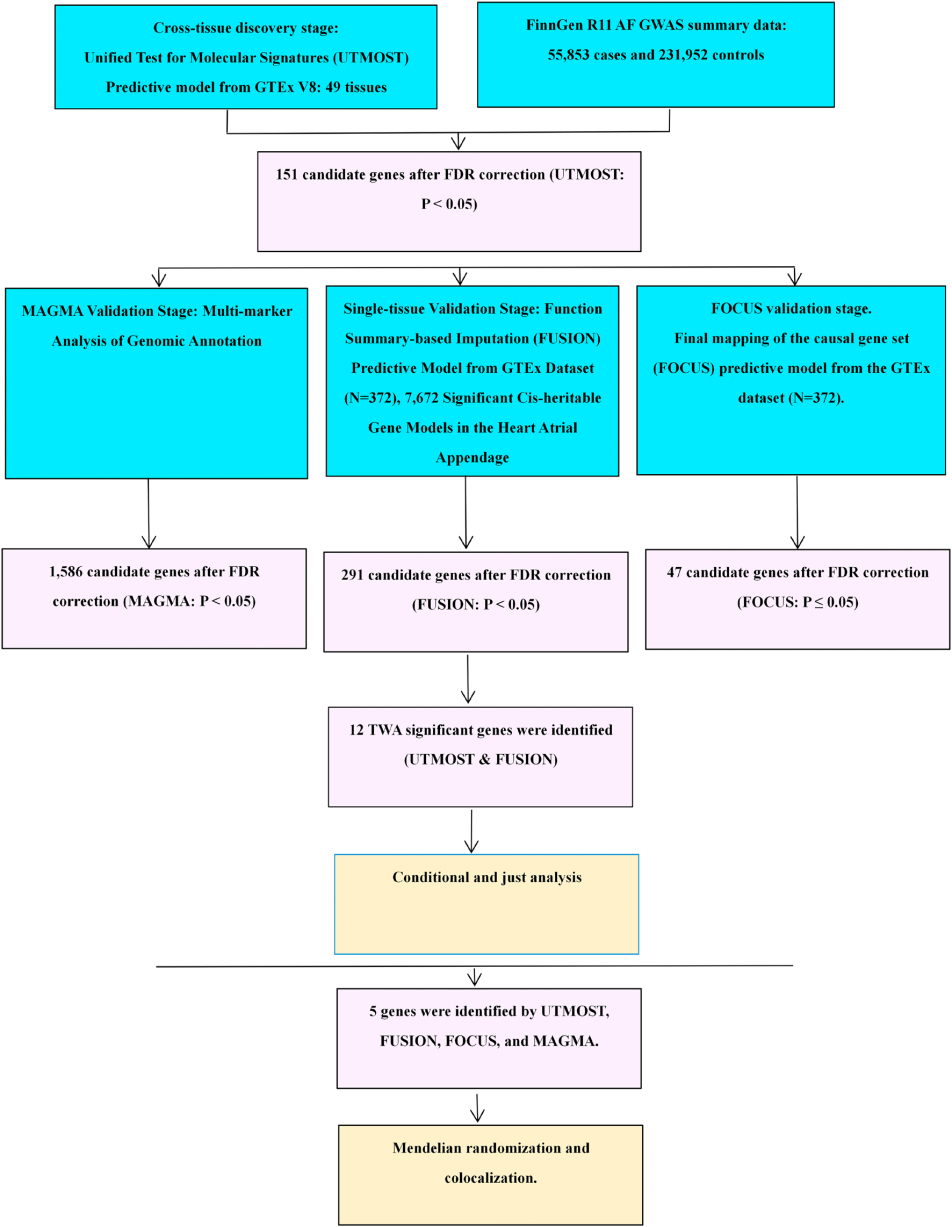
Overview of the TWAS framework for AF. FDR, false discovery rate; FOCUS, fine‐mapping of causal gene sets; FUSION, functional summary‐based imputation; GTEx, Genotype‐Tissues Expression Project; GWAS, genome‐wide association study; MAGMA, multi‐marker analysis of GenoMic annotation; TWAS, transcriptome‐wide association studies; UTMOST, unified test for molecular signatures.

The analytical process is depicted in Figure 1.

#### 2.1.2 AF GWAS data source

Our analysis integrated GWAS summary statistics from the FinnGen R11 dataset with expression quantitative trait loci (eQTL) data from the GTEx project. The FinnGen R11 dataset comprised 453 733 participants, including 55 853 AF cases and 231 952 controls, identified based on International Classification of Diseases codes. The GTEx V8 dataset provided gene expression data from 49 tissues, derived from post‐mortem samples of 838 individuals.

For replication analyses, we utilized an AF GWAS (Catalog accession code: ebi‐a‐GCST006414) comprising 1 030 836 European ancestry participants (60 620 cases; 970 216 controls), which represents the largest currently available AF GWAS summary statistics.

#### 2.1.3 eQTL files source

The GTEx V8 dataset encompasses a wealth of gene expression data from 49 different tissues (Table S1), collected from 838 post‐mortem donors (https://ftp.ebi.ac.uk/pub/databases/spot/eQTL/imported/GTEx_V8). The sample sizes varied across different tissues, ranging from 73 samples in the renal cortex to 706 samples in the skeletal muscle. The project ensures data quality and reliability by strictly selecting donors, with ages between 21 and 70 years, and tissue samples collected within 24 h post‐mortem. Exclusion criteria are minimal and include individuals who are HIV‐positive, engage in high‐risk behaviors, have viral hepatitis, metastatic cancer, received chemotherapy or radiotherapy in the past 2 years, had blood transfusions within 48 h, or have a BMI greater than 35 or less than 18.5.^11^ Notably, none of the donors were diagnosed with AF or other cardiovascular diseases. Therefore, the samples predominantly come from healthy individuals without major illnesses, and the clinical phenotype of the donor population is generally healthy.^11^ In the eQTL analysis, researchers carefully considered potential health biases. While a small number of donors may have had specific health conditions, these factors are unlikely to significantly impact the results of the TWAS.

### 2.2 Transcriptome‐wide association study (TWAS) analysis

In the discovery phase, the UTMOST methodology was utilized for cross‐tissue association testing.^5^ To evaluate gene‐trait correlations on an organismal level, this procedure included merging AF GWAS summary data with eQTL information from each of the 49 tissues included in the GTEx dataset. This technique aids in pinpointing genes significantly influencing trait heritability within tissues and refining the precision of these estimates.^5^ To combine gene‐trait relationships using the covariance of single‐tissue statistics, we next used the generalized Berk–Jones test.^12^ We established a statistically significant *P*_FDR_ threshold of <0.05 following a false discovery rate (FDR) adjustment.

The goal of merging AF GWAS data with eQTL data from the GTEx V8 atrial tissue using the FUSION tool was to reduce the likelihood of interference and false positives in cross‐tissue association testing.^13^ This analysis evaluated the links between candidate genes identified by UTMOST and AF risk within each tissue. Initially, linkage disequilibrium (LD) between SNPs and the prediction model was calculated using 1000 genome samples of European ancestry. To determine the overall effect of SNPs on gene expression weights, FUSION combined many prediction models (BLUP, BSLMM, LASSO, Elastic Net, and top 1). The model with the optimal predictive efficacy was chosen to calculate gene weights, which were then incorporated with the genetic effects on AF (AF GWAS *Z*‐score) for AF TWAS analysis. Candidate genes qualified for further analysis if they met the criteria of *P*_FDR_ < 0.05 in the cross‐tissue TWAS and *P*_FDR_ < 0.05 in at least one single‐tissue TWAS.

### 2.3 Conditional and joint analysis

Using the FUSION framework, we can isolate multiple relevant features from a small genomic region. We used genome‐wide (GW) FDR correction in conditional and combined analyses on the significant TWAS signals to find out if the genes at the same locus were independent or interconnected, following the steps outlined in reference.^14^ This method allows for the assessment of GWAS association signals after excluding the TWAS signals. Genes that retained significance post‐adjustment are designated as jointly significant genes, whereas those that failed to retain their significance are termed marginally significant genes, according to the classification outlined in reference.^15^

### 2.4 Focus analysis

To investigate the interrelationships among various traits linked to specific genetic locales, we utilized the fine‐mapping technique known as FOCUS.^16^ FOCUS is designed to identify the causal links of genetic variations (or genes) with complex traits by analyzing associations within a genomic region. This approach combines GWAS data with eQTL analyses and relies on the posterior inclusion probability (PIP) as a crucial metric. PIP assesses the probability that a particular genetic element influences the trait under consideration. A PIP value above 0.9 reflects a 90% confidence level that the genetic element plays a role in the development of the trait. Consequently, in this study, genes that achieved a posterior probability of 0.9 or greater were deemed significant. Generally, a heightened PIP indicates a robust likelihood of gene involvement in trait evolution. FOCUS not only improves the precision of pathogenic gene identification but also surpasses alternative methods in sensitivity, thus proving to be a superior tool for pinpointing disease‐associated genes.

### 2.5 MAGMA analysis

MAGMA begins by separating the SNP matrices of genes into their primary components. These components are subsequently utilized as variables in linear regression models that predict phenotypes. It next removes components with insignificant eigenvalues. This technique is notably effective for investigating polygenic traits and clarifying the functional and biological foundations of genetic elements within these traits.^17^, ^18^ Combining these approaches bolsters the reliability of the outcomes. Statistical significance was achieved by implementing the Benjamini‐Hochberg (B‐H) correction technique and setting a *P*_FDR_ threshold lower than 0.05.

### 2.6 MR and colocalization results

TWAS might be compromised by biases like horizontal pleiotropy and the presence of weak instrument variables, which could yield erroneous outcomes. MR, a gene‐centric method, aids in deducing causal impacts and directly assessing the variability between gene expression levels and phenotypes. In this MR analysis, we adhere to the three core assumptions of MR: (1) The instrumental variables (IVs) are strongly associated with gene expression, called the relevance assumption; (2) There are no unmeasured confounders affecting gene expression and AF, called the independence assumption; (3) The IVs affect AF only through gene expression, called exclusion restriction.^19^ This study followed the STROBE‐MR guidelines to enhance the reporting of epidemiological observational studies.

In this study, we employed two distinct significance thresholds (*p* < 5 × 10^−8^ and *p* < 1 × 10^−6^) for SNPs as IVs.^20^, ^21^ The GW significance threshold of *p* < 5 × 10^−8^ represents the conventional standard for genetic instrument selection in MR studies, ensuring compliance with the key relevance assumption of MR methodology. However, this stringent threshold may inadvertently exclude biologically important loci because of insufficient statistical power, particularly for genomic regions with limited instrument availability. To address this limitation and comprehensively evaluate potential causal associations between candidate genes and AF, we additionally implemented a suggestive significance threshold of *p* < 1 × 10^−6^, thereby enhancing our capacity to identify putative causal relationships while maintaining methodological rigor. LD clustering was performed to ensure SNP independence (*r* ^2^ < 0.3). SNPs with an F‐value of 10 or less were discarded to minimize the influence of weak instruments,^20^ and SNPs related to confounding variables were eliminated using PhenoScanner v2. To find and remove outliers, we used MR‐PRESSO, which stands for MR pleiotropy residual sum and outlier.^22^ After that, analysis could begin on the final dataset. We used the Wald ratio technique for cases with a single SNP, and the random‐effects IVW approach with weighted median (WM) and MR‐Egger regression as supplements for cases with multiple SNPs. For heterogeneity, we used the Cochran's *Q* test, and for horizontal pleiotropy, we used the MR‐Egger intercept test. The R package “TwoSampleMR”^23^ was used to do the analysis.

A possible genetic convergence between gene expression and illness manifestation could be indicated by the overlap of eQTL and GWAS data. “Coloc” was used to perform Coloc analysis, which provides posterior probabilities (PP) for different hypotheses, such as H0 (no causal variant), H1 (causal variant only in GWAS), H2 (causal variant only in eQTL), H3 (distinct causal variants for each trait), and H4 (shared causal variant).^24^ A PP4 value exceeding 75% denotes a high probability of a mutual causal influence on both traits. This analysis was conducted for the designated candidate genes.^6^, ^8^

### 2.7 GeneMANIA network analysis

To explore the functional implications of the identified genes, we performed GeneMANIA network analysis (https://genemania.org/).^25^ This approach constructs gene interaction networks based on co‐expression, physical interactions, and shared pathways, providing insights into the biological processes underlying AF susceptibility.

## 3 RESULTS

### 3.1 TWAS analyses in cross‐tissue and single tissue

In the cross‐tissue TWAS analysis, we identified 151 genes significantly associated with AF risk after FDR correction (*P*_FDR_ < 0.05) (Table S2). For single‐tissue validation, we assessed the association between the atrial appendage tissue and FUSION. In the GTEx dataset's atrial appendage tissue, 7626 genes showed significant cis‐regulated genetic expression, with 291 of these genes significantly associated with AF risk (*P*_FDR_ < 0.05) (Table S3). Figure S1 displays a Manhattan plot of the top 50 most significant genes after FDR correction. In both cross‐tissue and single‐tissue analyses, we identified 12 overlapping candidate genes using stringent thresholds (Table 1). All 12 genes are protein‐coding. Additionally, we successfully replicated all the significant loci reported by Hsu et al. (2018).^26^

**TABLE 1:** The significant genes for AF risk in both cross‐tissue and single‐tissue TWAS analysis.

Gene	BP0	BP1	Putmost	UTMOST_Discovery	FUSION_Replication	*Z*‐score	Pfusion	*P*_FDR_	Reported
				*P*_FDR_	Top GWAS ID				
CEP68	65 056 366	65 086 854	1.24E‐06	1.79 E‐04	rs1009358	7.12	1.07E‐12	5.44E‐10	Yes
FKBP7	178 463 664	178 478 600	2.54E‐11	9.03E‐09	rs890578	5.58	2.74E‐08	6.33E‐06	Yes
SPATS2L	200 305 881	200 482 263	4.36E‐07	7.09E‐05	rs159321	−5.37	7.76E‐08	1.60E‐05	No
UBE2D3	102 794 383	102 868 896	1.91E‐06	2.46E‐04	rs7694724	−4.80	1.57E‐06	2.39E‐04	No
SLC2A9	9 771 153	10 054 936	1.04E‐05	9.58 E‐04	rs4311315	4.55	5.32E‐06	6.65E‐04	No
CAMK2D	113 451 032	113 761 927	7.56E‐05	4.96 E‐03	rs4834352	4.23	2.28E‐05	2.26E‐03	Yes
CCNT2	134 918 235	134 959 342	1.52E‐05	1.21E‐03	rs1592	−3.87	1.08E‐04	7.10E‐03	No
SHROOM3	76 435 100	76 783 253	1.01E‐04	6.28E‐03	rs13148163	−3.77	1.61E‐04	9.82E‐03	No
CCDC141	178 830 269	179 050 086	7.03E‐09	1.64E‐06	rs3731746	−3.63	2.85E‐04	1.46E‐02	Yes
CCDC158	76 313 002	76 421 868	1.11E‐03	3.63E‐02	rs13148163	3.47	5.29E‐04	2.12E‐02	No
RPL9	39 454 124	39 458 586	6.41E‐04	2.64E‐02	rs2687963	3.29	1.01E‐03	3.28E‐02	No
FBXL5	15 604 539	15 681 661	3.10E‐04	1.57E‐02	rs7665390	3.12	1.79E‐04	4.82E‐02	No

Abbreviations: AF, atrial fibrillation; BP0, start base position; BP1, end base position; FUSION, functional summary‐based imputation; GWAS, genome‐wide association study; Reported, reported genes implicated in previous GWASs; Top GWAS ID, rsID of the most significant GWAS SNP in the locus; TWAS, transcriptome‐wide association study; UTMOST, unified test for molecular signatures; Yes/No, previously reported in AF GWAS.

### 3.2 Conditional and joint analysis

To assess the independence of association signals within each genomic locus, we performed conditional and joint analyses. These analyses were designed to determine whether the identified genes independently contribute to AF risk or if their signals are driven by shared regulatory mechanisms. We identified 11 distinct loci harboring multiple genes significantly associated with AF risk (*p* < 0.05), including CEP68 (2p14), FKBP7 (2q31), SPATS2L (2q33.1), UBE2D3 (4q24), CAMK2D (4q26), SLC2A9 (4p16.1), SHROOM3 (2q31.2), CCDC158 (4q21.1), CCNT2, RPL9, and FBXL5 (Table S4).

Notably, FKBP7 emerged as a key contributor to the signal at the 2q31.2 locus. When we adjusted for the predicted expression of FKBP7, the TWAS signal for CCDC141 was significantly attenuated, suggesting that FKBP7 plays a central role in modulating this association (Figure S2). This finding implies that FKBP7 may act as a regulatory hub within this genomic region, potentially influencing the expression of neighboring genes and thereby contributing to AF risk.

Following conditional analysis, the association signals for the 11 genes remained significant, indicating that these genes are independently associated with AF risk and that their signals are not merely because of LD within the locus. This underscores the robustness of these genetic associations and highlights the importance of considering gene‐specific effects in the genetic architecture of AF.

### 3.3 Focus analysis

LD between SNPs and the coordinated regulation of gene models can affect the accuracy of identifying AF‐related genes through TWAS. To mitigate this issue, we utilized FOCUS to enhance the assessment of gene probabilities based on predicted modules, recommended LD reference data, and AF GWAS summary statistics. FOCUS identified 47 genes with a significant causal association with AF (Table S5).

### 3.4 MAGMA analysis

Using the input AF GWAS data, we performed MAGMA gene‐based analysis to identify genes significantly associated with AF risk. After FDR correction, 1586 genes were found to be significantly associated with AF (*P*_FDR_ < 0.05) (Table S6, Figure S3).

To further explore the biological relevance of these genes, we conducted pathway enrichment analysis. The top three enriched pathways were related to: (1) the contraction process of myocardial cells (GO:0009888), (2) the regulation of heart contraction (GO:0008016), and (3) the conduction of cardiac electrical signals (GO:0060047) (Table S7, Figure S4). These pathways are critical for maintaining normal cardiac function, and their enrichment highlights the importance of myocardial contractility and electrical activity in AF pathogenesis.

Additionally, tissue‐specific MAGMA analysis revealed that the SNPs most significantly enriched for AF‐associated genes were localized in the Heart Atrial Appendage (Figure S5). This finding aligns with the anatomical and functional relevance of the atrial appendage in AF, as it is a key site for the initiation and maintenance of arrhythmias. The tissue‐specific enrichment further supports the hypothesis that genetic variants influencing atrial‐specific gene expression play a pivotal role in AF susceptibility.

### 3.5 Comparison of TWAS for different genetic approaches

**FIGURE 2:**
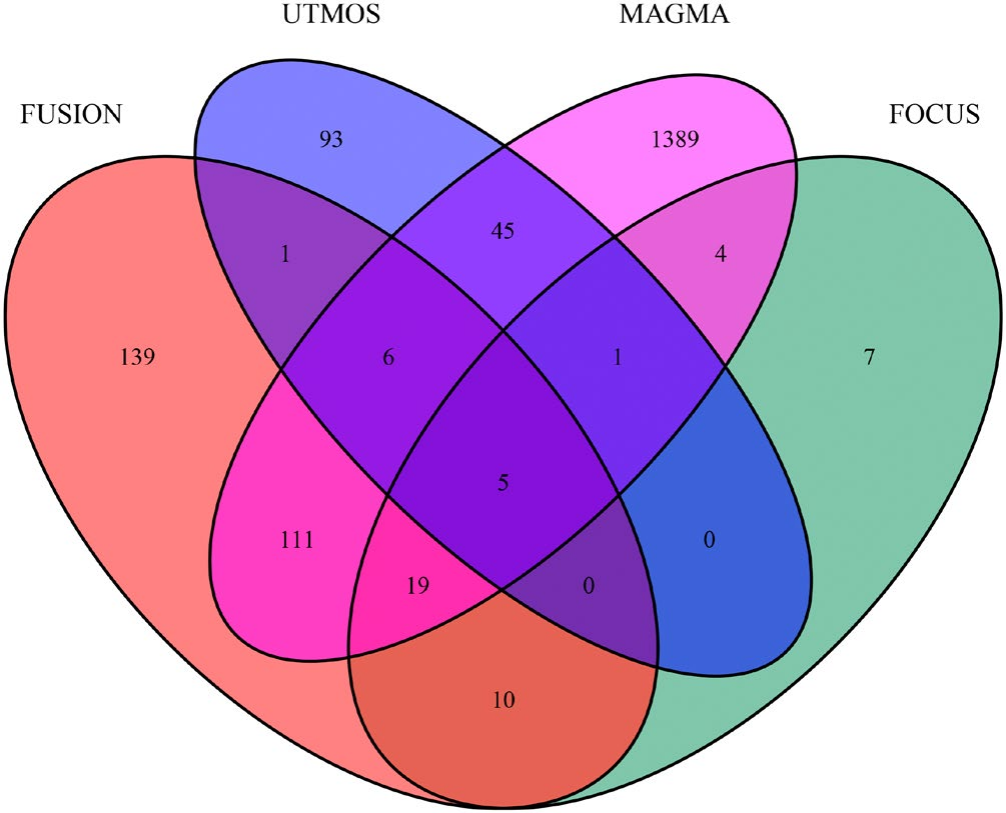
Venn diagram. MAGMA identified 1586 significant genes related to atrial fibrillation, FUSION pinpointed 291, and UTMOST cross‐tissue analysis highlighted 151. FOCUS detected 47, among which 5 were common.

To validate the robustness of our findings, we compared the significant genes identified by UTMOST cross‐tissue analysis with those detected by FUSION, MAGMA, and FOCUS (Figure 2). Among the candidate genes, five genes—CEP68, FKBP7, SPATS2L, CAMK2D, and SHROOM3—emerged as the most robust candidates, with TWAS p‐values that remained significant after multiple testing corrections. Notably, SPATS2L and SHROOM3 were identified as novel susceptibility genes, not previously reported in the context of AF.

The consistency of these genes across multiple analytical approaches underscores their reliability as potential contributors to AF risk. The identification of SPATS2L and SHROOM3 as novel candidates highlights the power of integrating cross‐tissue TWAS with complementary methods to uncover new genetic insights into AF pathogenesis.

### 3.6 MR and coloc results

**FIGURE 3:**
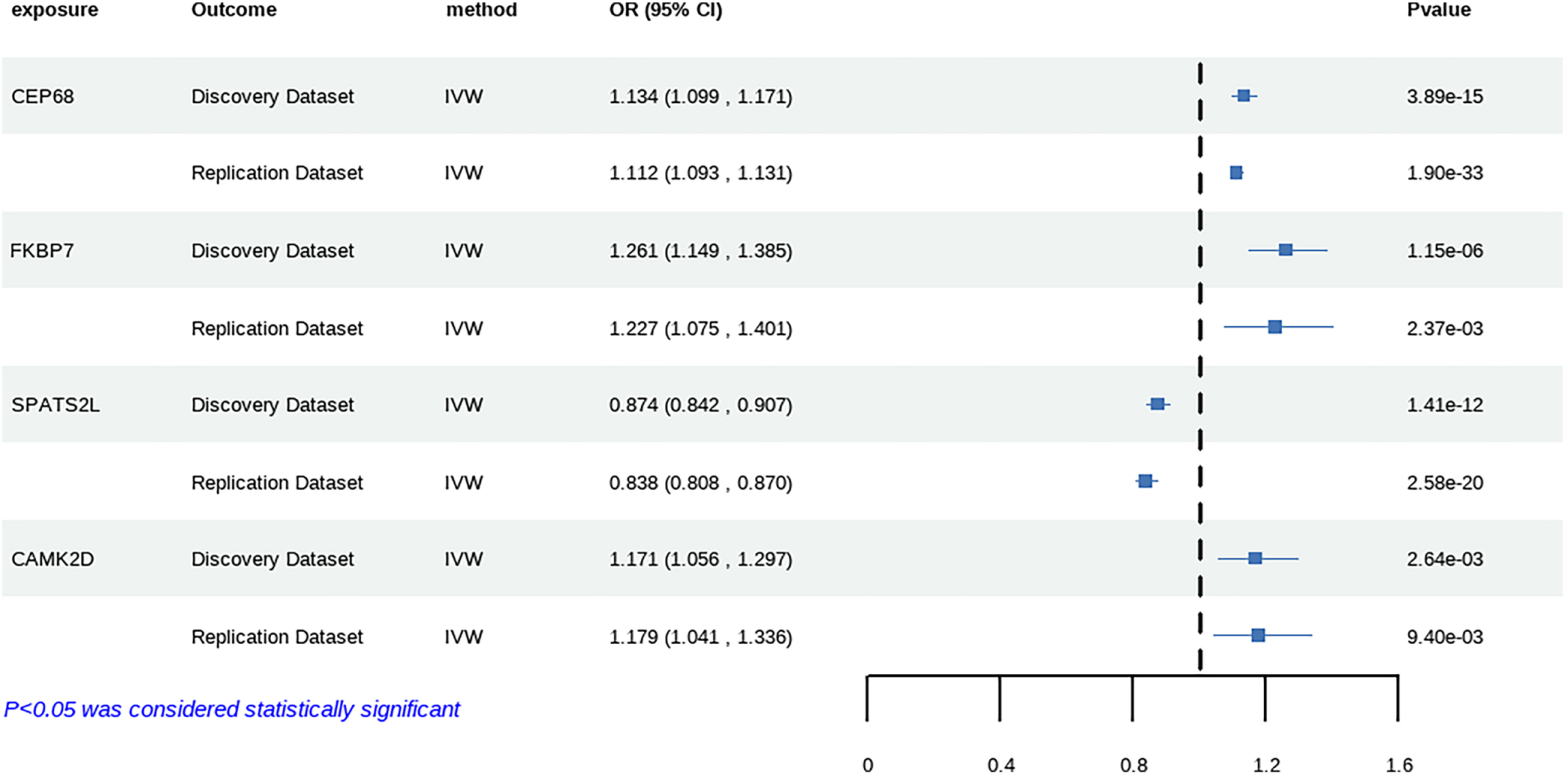
Further MR analysis confirmed the causal associations between these four genes and AF. CI, confidence interval; IVW, inverse‐variance weighted; OR, odds ratio.

The two‐sample MR analysis utilized eQTL data from GTEx and two AF GWAS datasets. While no valid IVs were identified for SHROOM3, the analysis revealed significant causal associations between AF and four other genes. MR results demonstrated that increased expression of the SPATS2L gene was associated with reduced AF risk (odds ratio [OR] = 0.874; 95% confidence interval [CI], 0.842–0.907; *p* = 1.41 × 10^−12^). Conversely, elevated expression of FKBP7 showed a positive association with AF susceptibility (OR = 1.261; 95% CI, 1.149–1.385; *p* = 1.15 × 10^−6^). Similarly, higher expression levels of CEP68 (OR = 1.134; 95% CI, 1.099–1.171; *p* = 3.89 × 10^−15^) and CAMK2D (OR = 1.171; 95% CI, 1.056–1.297; *p* = 2.64 × 10^−3^) were significantly associated with increased AF risk (Figure 3, Table S8). These findings were consistently replicated in an independent validation dataset (Figure 3, Table S8). Sensitivity analyses showed no evidence of heterogeneity or horizontal pleiotropy for any genes except FKBP7, which exhibited heterogeneity in the replication cohort (Table S8).

**FIGURE 4:**
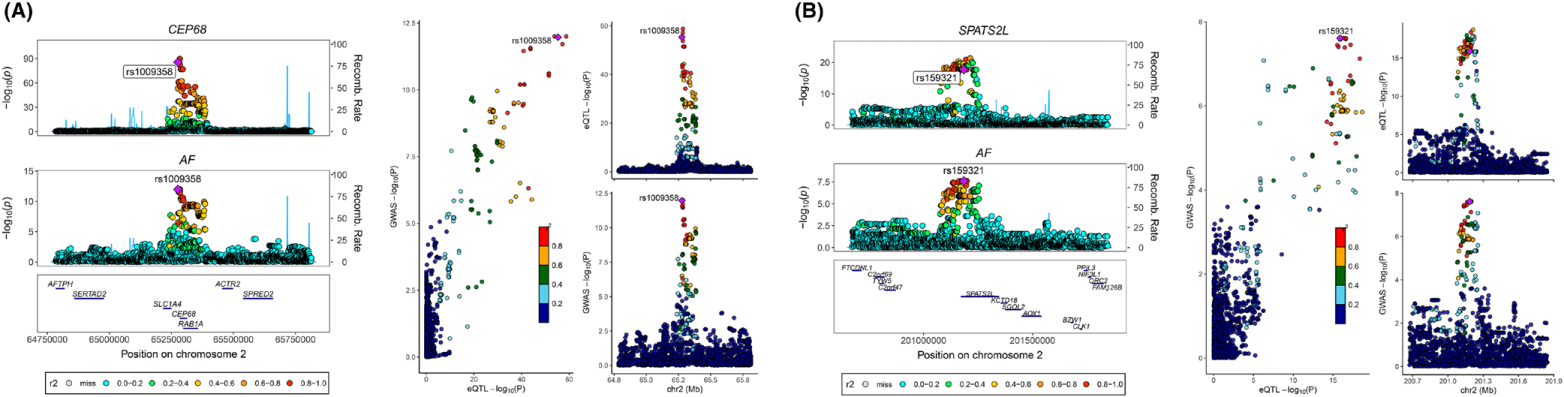
The results of Coloc analysis between candidate genes and AF. Colocation of eQTL and GWAS associations for CEP68 (A) and SPATS2L (B). A scatterplot highlights the convergence of GWAS and eQTL associations for these genes.

We conducted a coloc analysis on four reliable candidate genes to assess the probability of shared signals between GWAS and eQTL. The colocalization analysis demonstrated spatial overlap of GWAS and eQTL signals at both the *CEP68* and *SPATS2L* loci. The *CEP68* locus exhibited a high‐confidence colocalization signal (PP4 = 0.99), where the lead SNP rs1009358 and SNPs within its strong LD region showed significant associations with AF risk (Figure 4, Table S9). Similarly, the *SPATS2L* locus reached the statistical threshold for colocalization (PP4 = 0.87), with variants in the LD block containing the core SNP rs159321 displaying strong associations with AF phenotypes (Figure 4, Table S9). These findings suggest potential causal relationships.

### 3.7 Gene MANIA analysis

**FIGURE 5:**
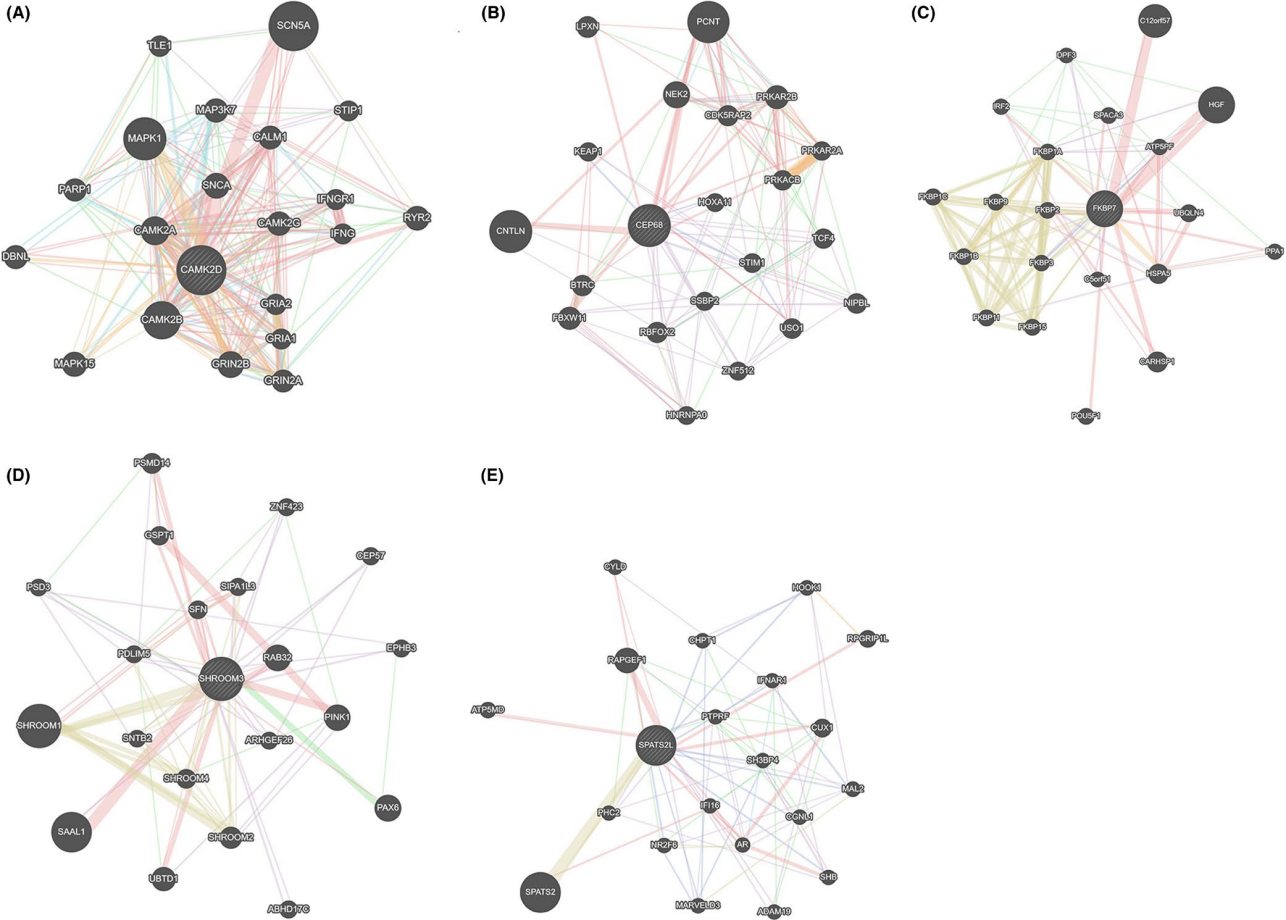
GeneMania gene network. (A) CAMK2D as the core, (B) CEP68 as the core, (C) FKB7 as the core, (D) SHROOM3 as the core, and (E) SPAST2L as the core.

Figure 5 shows the possible interaction gene network constructed around CAMK2D, CEP68, FKBP7, SHRMOO3 and SPAST2L for the central component. The regulation of cation transmembrane transport, regulation of ion transmembrane transport, and regulation of transporter activity are the three most important functional pathways enriched in CAMK2D‐related gene networks (Figure 5A and Table S10) cell cycle G2/M phase transition, response to glucagon, and organelle localization by membrane tethering are the three most important functional pathways enriched in CEP68‐related gene networks (Figure 5B and Table S10). The most significant functional pathways enriched in FKBP7‐related gene networks are drug binding, protein folding, and cis‐trans‐isomerase activity (Figure 5C and Table S10). The most significant functional pathways enriched in SHROOM3‐related gene networks are actin cytoskeleton, pigment accumulation, and cell–cell junction (Figure 5D and Table S10). However, for the network centered on SPATS2L (Figure 5E), there are currently no significant functional pathways enriched in SPATS2L‐related gene networks, indicating that further research is needed to confirm its role and relationship with AF in the future.

## 4 DISCUSSION

AF is a prevalent cardiac arrhythmia with a complex genetic architecture, and despite extensive research, its underlying mechanisms remain incompletely understood. Although GWAS have identified over 100 AF risk loci, the functional mechanisms that drive these associations are largely unknown. TWAS help bridge this gap by integrating gene expression data with genetic variation, offering valuable insights into disease pathogenesis. In this study, we employed a cross‐tissue TWAS approach, combining GWAS data from FinnGen R11 with multi‐tissue eQTL data from GTEx V8. This allowed us to identify five susceptibility genes—CEP68, FKBP7, SPATS2L, CAMK2D, and SHROOM3—associated with AF risk. Notably, SPATS2L and CEP68 emerged as novel candidates, with SPATS2L being reported for the first time in the context of AF.

SPATS2L (also known as SGNP or DNAPTP6) is a broadly expressed gene localized to human chromosome 2q33.1, with activity observed in 25 tissues, including the heart and gallbladder. Previous studies have demonstrated that this gene regulates cell growth through ribosomal pathways and modulates cell proliferation under oxidative stress by influencing ribosome biogenesis and translational control in myoblasts.^27^ Notably, the SNP rs295140 near the SPATS2L locus has been significantly associated with shortened QT intervals. Functional annotation revealed potential interactions between SPATS2L and SCN5A (a key gene encoding the cardiac sodium channel) via protein–protein interaction networks, suggesting a synergistic role in maintaining myocardial ion homeostasis.^28^ Dysfunction of SCN5A, extensively linked to AF pathogenesis in prior studies, provides a theoretical basis for SPATS2L's indirect involvement in arrhythmogenesis.^29^

Integrative multi‐omics analyses further identified significant genetic correlations between SPATS2L and AF risk factors such as hypertension and diabetes. MR and colocalization analyses in this study suggest that elevated SPATS2L expression may reduce AF risk through shared causal variants, implying a potential protective effect against AF development. However, the biological mechanisms underlying these associations remain unclear, particularly whether SPATS2L influences AF via SCN5A‐dependent pathways or independent regulatory cascades. Future studies should employ functional experiments (e.g., gene editing and ex vivo cardiomyocyte models) and animal models to validate SPATS2L's direct effects on cardiac electrophysiology and elucidate its interplay with sodium channel activity, calcium homeostasis, and other pathological processes critical to AF.

The 68 kDa centrosomal protein (CEP68), a key gene encoding a centrosome‐localized protein, plays an essential role in microtubule nucleation and spindle formation during cell division.^30^ MR and colocalization analyses in this study demonstrated a significant association between elevated CEP68 expression and an increased risk of AF. This finding aligns with prior genetic research, where transcriptomic profiling of left atrial appendage tissues from AF patients revealed CEP68 involvement in AF progression.^26^ Furthermore, a recent genetic analysis identified CEP68 as a shared candidate gene between AF and sinus node dysfunction, with AF‐associated SNPs enriched in cardiac tissues, underscoring its potential role in arrhythmia pathways. Despite accumulating genetic evidence, the precise mechanisms by which CEP68 influences AF remain speculative. Causal relationships and tissue‐specific pathways require rigorous validation.^31^ For instance, whether CEP68 impacts AF through direct regulation of ion channels, calcium handling, or via structural remodeling (e.g., fibrosis) has yet to be explored. To address this gap, future studies should employ conditional knockout models to evaluate CEP68's effects on cardiac electrophysiological parameters (e.g., action potential duration, conduction velocity) and assess its interaction with established arrhythmogenic pathways.

FKBP7, located on human chromosome 2q31.2, encodes the protein FKBP7, also known as FKBP23 or PPI ase (peptidylprolyl isomerase). This protein plays several crucial roles in the body, including accelerating protein folding and functioning as a molecular chaperone, which is vital for maintaining cellular protein homeostasis and physiological functions.^32^ FKBP7 is widely expressed across various human tissues, including the ovary and endometrium. In GWAS led by Jeffrey Hsu, Shamone Gore‐Panter, and their team, FKBP7 was pinpointed as a susceptibility gene for AF through RNA sequencing of left atrial appendages from a diverse cohort of 265 individuals.^26^ This finding aligns with our research results, further supporting the role of FKBP7 in AF susceptibility.

Located on chromosome 4q26 in humans, CAMK2D encodes for calcium/calmodulin‐dependent protein kinase II delta, which belongs to the serine/threonine protein kinase family and falls under the calcium/calmodulin‐dependent kinase subgroup. It primarily governs calcium signaling^33^ and is profusely expressed across the heart, brain, and 24 additional tissues. Research shows that CAMK2D plays a role in modulating NF‐κB signaling in adult cardiac fibroblasts.^34^ Elevated levels and activity of CaMKII are associated with heart failure,^35^ while its overexpression can influence the excitation‐contraction dynamics in cardiac and isolated muscle cells.^36^, ^37^ Experimental evidence suggests that CaMKII acts as a molecular signal connecting increased oxidative stress to AF, and strategies to reduce oxidative CaMKII may help prevent or reduce AF.^38^, ^39^ Our study reaffirms a significant causal link between CAMK2D expression and elevated AF risk.

SHROOM3, located on human chromosome 4q21.1, stands for SHROOM3 family member 3. It encodes a protein containing a PDZ domain and belongs to the shroom‐related protein family. This gene plays an important role in regulating cell shape and neuronal development.^40^ Mutations in SHROOM3 have been linked to various diseases and pathological conditions, suggesting potential clinical significance. Furthermore, research has shown that genetic variations within SHROOM3 are associated with chronic kidney disease. This association extends to changes in known kidney disease markers, including baseline estimated glomerular filtration rate, urine albumin‐to‐creatinine ratio, and blood urea nitrogen levels.^41^ Unfortunately, MR did not identify suitable instrumental variables representing SHROOM3 gene expression; this might be because of factors such as sample size limitations. Further experimental evidence is needed to explore the relationship between SHROOM3 and AF.

Approaches like cross‐tissue TWAS, single‐tissue TWAS with subsequent gene analysis validation, and Coloc MR are widely adopted to pinpoint susceptibility genes across various diseases. A recent cross‐organizational TWAS study identified two new susceptibility genes for migraine,^6^ employing a design similar to our study. In our research, to overcome the challenges posed by single‐tissue analysis, we implemented a cross‐tissue strategy to identify more dependable gene candidates. Several TWAS‐significant genes located in known AF loci have been robustly validated in previous functional studies. For example, increased reactive oxygen species in CAMK2D are associated with AF,^38^, ^39^ promoting its onset. This finding aligns with our research. Moreover, as the GTEx project data expands and stricter significance thresholds are applied, our study is expected to yield even more stable and accurate results.

Future research will combine in vitro and in vivo functional experiments, clinical data analysis, and real‐world data to further validate the function and clinical potential of these newly identified susceptibility genes for AF. Preclinical validation typically involves gene knockout or overexpression models in mice, as well as organoid systems, which can be used to investigate the role of these genes in the pathogenesis of AF.^42^ Clinical validation is carried out through retrospective patient data analysis, clinical trials, and biomarker correlation studies, which help assess the association between genes and AF, as well as predict their therapeutic responses.^43^, ^44^ Additionally, real‐world data, such as electronic health records and biobank resources, provide valuable support for validating the clinical relevance of these genes across different populations.^45^

### 4.1 Strengths of the study

While GWAS have identified numerous robust SNPs and susceptibility loci associated with AF,^46^ significant challenges persist in establishing causal links between these loci and their underlying pathophysiological pathways. Their noncoding localization (>90%) complicates functional interpretation. TWAS overcomes this limitation by prioritizing gene‐level associations linked to expression quantitative trait loci (eQTLs), bridging noncoding variants to regulatory mechanisms.^47^ Although whole‐exome sequencing (WES) reveals rare coding variants (e.g., CTNNA3 and GATA4),^32^ it neglects 98% of noncoding regulatory elements addressed by TWAS, which systematically connects these regions to gene expression perturbations underlying AF pathogenesis.

In this study, we employed a cross‐tissue TWAS analysis approach, integrating gene expression data from 49 tissues, including both cardiac and non‐cardiac tissues. This strategy enhances statistical power by leveraging shared genetic regulatory mechanisms across tissues, increasing the effective sample size, particularly for tissues that are difficult to obtain.^33^, ^34^, ^35^, ^36^ It also provides a more comprehensive understanding of genetic influences, as many eQTLs with large effects regulate gene expression across multiple tissues,^37^ and substantial sharing of local expression regulation has been observed.^38^ By incorporating cross‐tissue data, we consider a broader regulatory landscape that may influence AF susceptibility, rather than restricting the analysis to cardiac tissues alone, thereby avoiding the omission of important genetic signals that may manifest in non‐cardiac tissues. Furthermore, jointly modeling multiple genetically correlated tissues reduces the burden of multiple testing and improves the accuracy of association results. The issue of statistically significant associations appearing in tissues not directly related to the trait has been widely discussed, but multi‐tissue approaches have been shown to enhance eQTL detection^33^, ^34^, ^35^, ^36^ and improve statistical power in genetic studies of complex traits.^39^, ^40^, ^41^ Since AF is a complex trait that may be influenced by systemic genetic factors, limiting the analysis to cardiac tissues alone could overlook crucial regulatory effects. Our cross‐tissue approach provides a more comprehensive characterization of AF‐related genes, offering new insights into its genetic architecture.

### 4.2 Limitations of the study

This study has several limitations. First, while our cross‐tissue TWAS‐MR framework identified SPATS2L and CEP68 as potential AF susceptibility genes through functional prioritization, the molecular mechanisms underlying these associations require experimental validation using cellular models and in vivo systems. Second, reliance on European‐biased genomic datasets (FinnGen/GTEx v8) limits population generalizability, necessitating multi‐ethnic replication for robust clinical translation. Third, despite rigorous statistical adjustments, inherent technical constraints persist regarding tissue‐specific eQTL effect variability and expression prediction accuracy, which could introduce residual confounding. Future studies should prioritize functional characterization of candidate genes in AF‐relevant cardiac tissues and expand ancestral diversity in genomic resources to strengthen causal inference and clinical applicability.

## 5 CONCLUSION

This study identified SPATS2L, CEP68, FKBP7, CAMK2D, and SHROOM3 as novel genetic contributors to AF, with SPATS2L and CEP68 representing unprecedented discoveries. By integrating cross‐tissue TWAS with MR and colocalization, we highlighted the interplay between transcriptional regulation and AF susceptibility. Future work should give priority to the functional verification of in vitro and in vivo models, as well as the multi‐ethnic cohort to enhance the relevance of translation.

## AUTHOR CONTRIBUTIONS

BL, YY, and XZ designed the study. YY, WZ, and ZR performed the data analysis. YY and WZ drafted the article. XZ and YY collected the data. BL reviewed and provided final approval. All authors contributed to the article and approved the submitted version.

## FUNDING INFORMATION

Funding for this project was provided by the National Natural Science Foundation of China (grant no. 81970391).

## CONFLICT OF INTEREST STATEMENT

Authors declare no conflict of interests for this article.

## ETHICAL APPROVAL AND CONSENT TO PARTICIPATE

Ethical review and approval were not required for the study on human participants in accordance with local legislation and institutional requirements. Written informed consent was not required to participate in this study in accordance with local legislation and institutional requirements.

## Supporting information


Figure S1.
Figure S2.
Figure S3.
Figure S4.
Figure S5.

Table S1.

Table S2.

Table S3.

Table S4.

Table S5.

Table S6.

Table S7.

Table S8.

Table S9.

Table S10.

## Data Availability

The datasets generated during and/or analyzed during the current study are available in the FinnGen study repository [https://www.finngen.fi/en/access_results]. Gene expression and eQTL data are freely available at https://ftp.ebi.ac.uk/pub/databases/spot/eQTL/imported/GTEx_V8.
