# Supplementary material for: A cross‐tissue transcriptome‐wide association study identifies novel susceptibility genes for atrial fibrillation
Source: J Arrhythm. 2025 May 22;41(3):e70097. doi: 10.1002/joa3.70097 (PMC12099065; doi:10.1002/joa3.70097)
Supplement: Supplementary file 1 — Figure S1. Figure S2. Figure S3. Figure S4. Figure S5. [file JOA3-41-e70097-s003.docx]

| 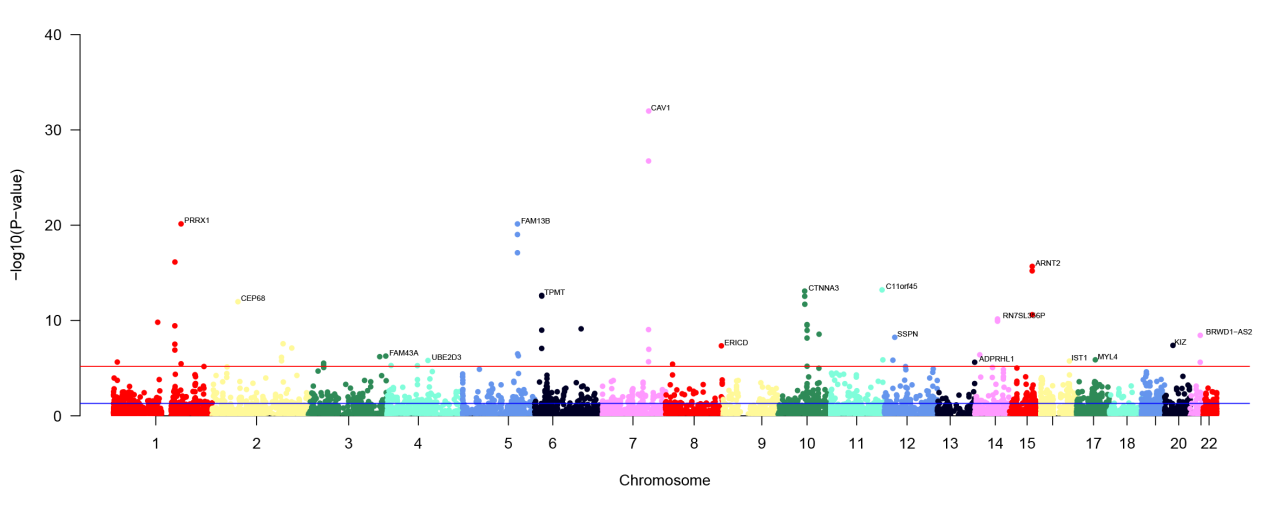 |
| --- |
| Figure S1. The Manhattan plot results from a single-tissue TWAS for AF.  50 most significant genes after FDR correction specifically associated with the risk of AF. The y-axis represents pvalue in –log (10) scale. |

| 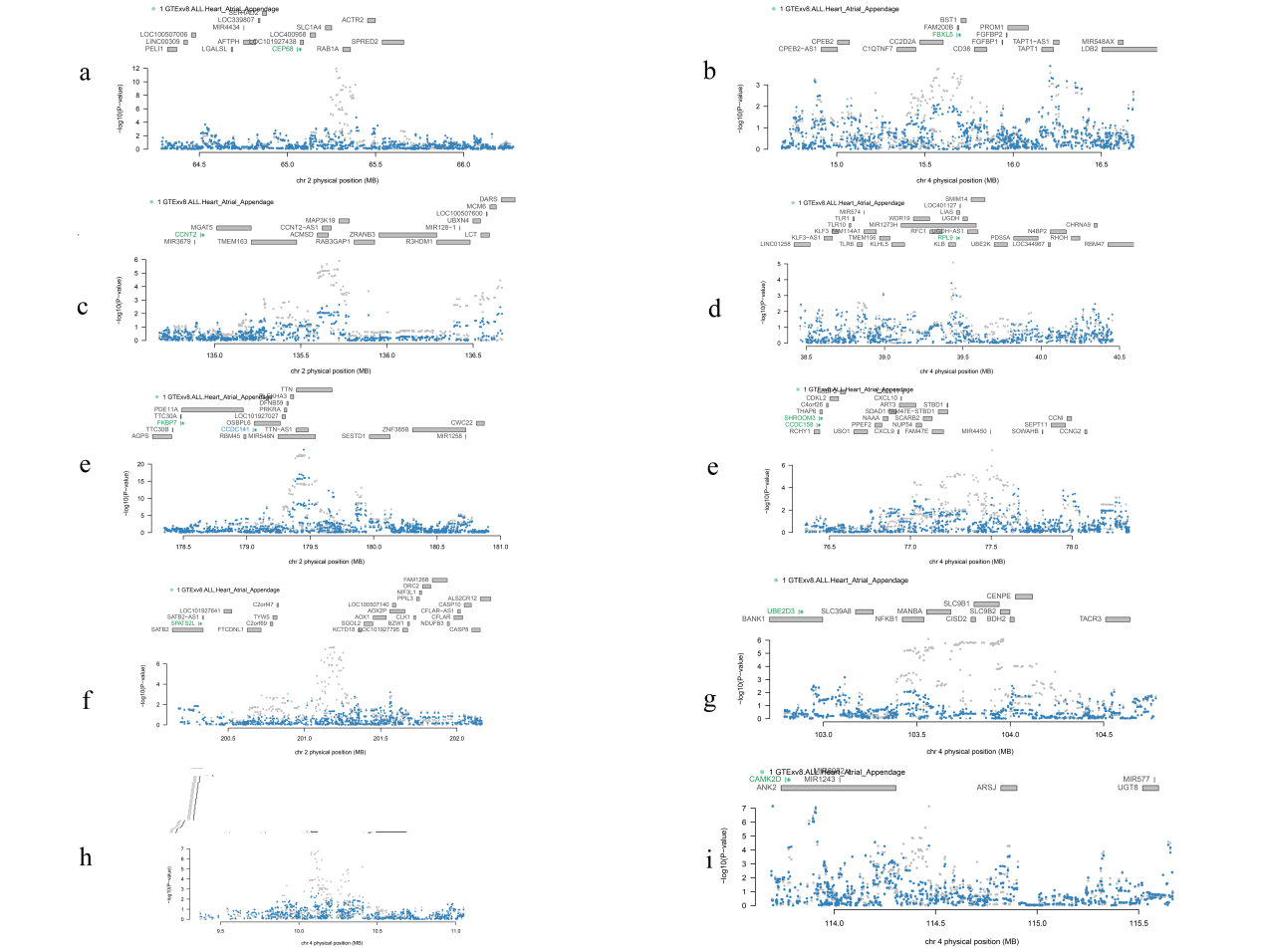 |
| --- |
| Figure S2. Regional association of TWAS hits.  (a, b, c, d) Chromosome 2 regional association plots. (e, f, g, h, i, j) Chromosome 4 regional association plots. Each plot’s upper panel showcases all genes within that region. TWAS-associated edge genes are indicated in blue, while genes that are conditionally significant appear in green. The upper panels illustrate regional Manhattan plots of GWAS data, contrasting the conditions before (grey) and after (blue) accounting for the expression of the green genes. |

| 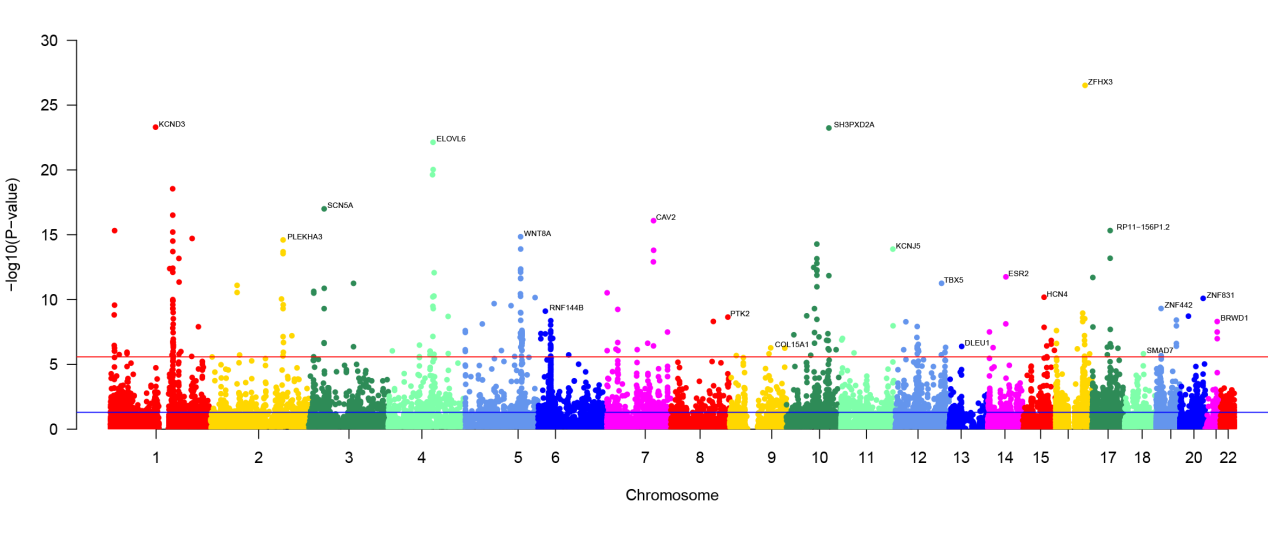 |
| --- |
| Figure S3. Manhattan plot from a MAGMA gene-based analysis for AF  This gene-based assessment, conducted via MAGMA, was based on input GWAS summary statistics. Red lines mark the levels of GW significance (−log10 P). |

| 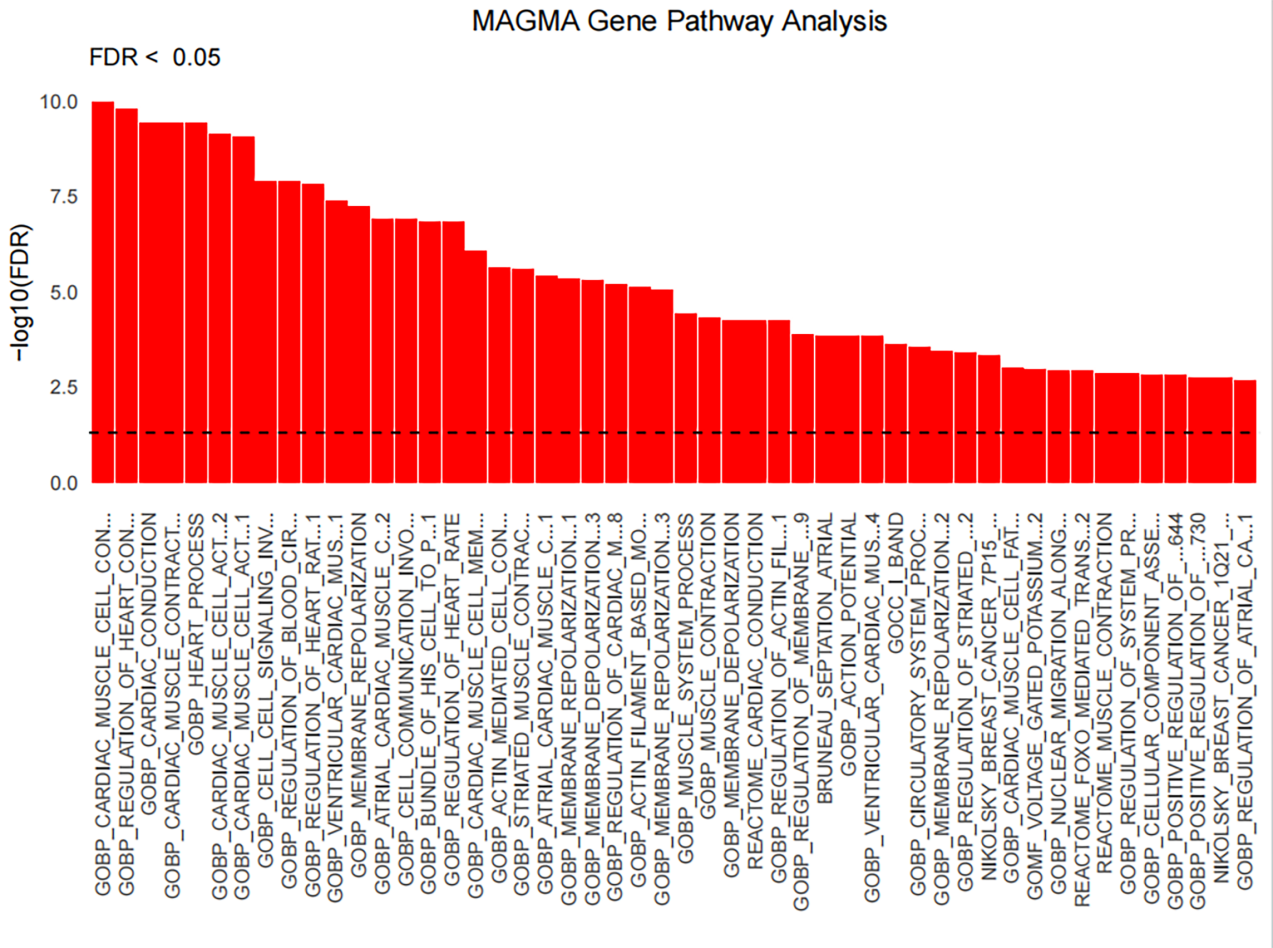 |
| --- |
| Figure S4. Significant types of pathways in terms of the GO and KEGG enrichment analyses through KEGG. BP, biological process |

| 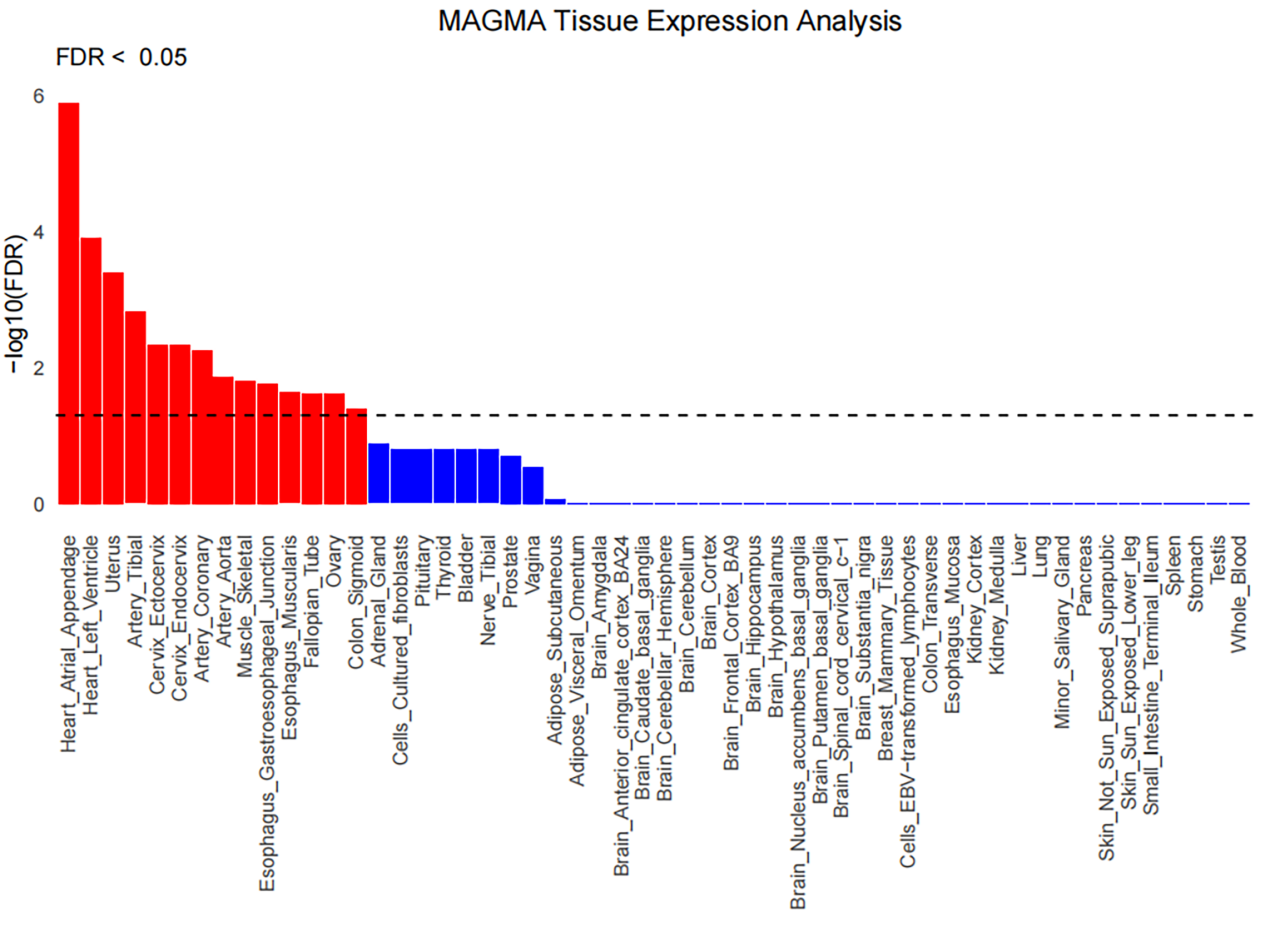 |
| --- |
| Figure S5. Tissue specific enrichment of SNP heritability for AF through MAGMA.  P values are shown in the y-axis with a scale of − log10. The bars in black represent significant enrichment with FDR adjustment for multiple hypothesis testing. |
